# Investigating the predicting role of COVID-19 preventive measures on building brand legitimacy in the hospitality industry in Tanzania: mediation effect of perceived brand ethicality

**DOI:** 10.1186/s43093-022-00128-6

**Published:** 2022-06-08

**Authors:** David Amani, Ismail Juma Ismail

**Affiliations:** Department of Business Administration and Management, The University of Dodoma, Dodoma, Tanzania

**Keywords:** COVID-19 preventive measures, Brand legitimacy, Perceived brand ethicality, Hospitality industry

## Abstract

The COVID-19 pandemic undesirably affected the hospitality industry, and therefore, preventive measures have been advocated as crucial when revitalizing or rejuvenating the industry. This study investigated the interplay of predicting role of COVID-19 preventive measures, perceived brand ethicality, and brand legitimacy in the hospitality industry in Tanzania during the period of reviving the industry. Furthermore, the study examines the mediating role of perceived brand ethicality in the relationship between COVID-19 preventive measures and brand legitimacy. Data were collected from a total of 405 customers of hospitality organizations recruited via an on-site survey. Data analyses were done using structural equation modeling. Overall, the results have shown that COVID-19 preventive measures had a direct positive effect on brand legitimacy. Additionally, COVID-19 preventive measures could enhance brand legitimacy indirectly via perceived brand ethicality. The study has significant implications for different hospitality organizations and operators in Tanzania and other countries during post the COVID-19 period.

## Introduction

Recently, there have been scholarly efforts to conceptualize a brand as an entity constructed, experienced, and shaped by specific social communities to build legitimation [5, 66]. Legitimacy is a precondition of value creation since a brand that has not been legitimized is unlikely to be positively appraised by customers [5, 28]. References [13, 32] argued that legitimacy is an important mechanism in shaping and reshaping the different subcultures’ purchase and consumption meanings and practices. Customers make judgments about the decision to buy and consume a certain brand, whether it is righteous or ridiculous [28]. Overall, a righteous form of customer’s purchase and consumption indicates legitimation of the brand [37]. It denotes customers' perceptions or assumptions that business organizations' practices are desirable and correct within a specific social constructed structure of norms, values, beliefs, and definitions [28]. At the societal level, overall judgment about legitimacy is determined by the legal-regulatory framework, moral standards, and social norms [63]. Therefore, it is agreed that brand legitimacy is granted by members of institutional environments, including business partners, government agencies, and, more importantly, customers [21].

Numerous studies have been conducted to examine how different products build legitimacy, including cultural products and cultural heritage products [62, 66], marketing activities [7] and higher education institutions [5, 43]. Furthermore, other studies examine how business organizations' routine practices and operational capabilities can affect their legitimacy [24]. Thus, building legitimacy has now been considered the most important dimension for long-lasting business success. Reference [49] suggests that business organizations require legitimacy to acquire social acceptance, an important intangible asset for business organizations that strive to survive and grow in turbulent situations in the marketplace. In a turbulent situation in the marketplace, social acceptance is of paramount importance for any business organization since it connects it to an acceptable system of norms, beliefs, and opinions [3, 19, 66]. Based on this argument, several scholars have recommended more studies on how legitimacy is constructed among business organizations [8]. Recent studies have further recommended that legitimacy is an intangible resource that could help service organizations, such as those in the hospitality industry, operate in situations that involve an interplay between social crisis and business performance [51].

Empirical studies show that the growing interest among scholars in examining how customers grant legitimacy to business firms and their brands is due to the business climate, which includes characteristics such as cynicism, boycotts, ethical purchase, and ethical consumption [30, 55]. Recent studies have revealed the need to examine how customers can grant legitimacy to business organizations during pandemic situations such as COVID-19 [22, 47]. It is widely accepted that business organizations could demonstrate their accountability and responsibility for social welfare or well-being during pandemics to build social acceptance [4, 8, 15]. Overall, legitimacy refers to practices that promote ethical practices in order to maximize profits while focusing on or emphasizing the social well-being or social welfare of current and potential customers [47]. It is agreed that customer climates similar to cynicism can be addressed through approaches that enhance business organizations' willingness to consider social welfare and wellbeing, particularly during pandemics [2, 50]. Therefore, it is agreed that scholarly understanding of how customers grant legitimacy or social acceptance to business organization is undoubtedly significant to marketers and scholars [49, 67]. Through negotiations, marketers must align marketing efforts with social norms, and cultural issues to ensure customers ascribe meaningful meaning to the brands [28]. When looking at pandemics like COVID-19 in the hospitality industry, researchers have found that becoming more credible can help offset the negative effects of COVID-19, like a big drop in the number of customers [47]. Understanding customer legitimacy has thus proven useful for hospitality organizations seeking to build trust during and after the COVID-19 pandemic [67]. Overall, this study found that hospitality organizations get their legitimacy from certain institutional environments in which customers are important parts of this environment.

Empirical evidence suggests that brand legitimacy should be perceived as a value co-creation process. Therefore, customers as value co-creators have recently been included in institutional environments as legitimacy-granting constituents [67]. Although customers have been categorized as important members of institutional environments, limited studies have investigated customers as legitimacy-granting constituents [15, 37]. Scholars such as [37] argue that the significant role of the legitimacy-based institutional atmosphere in the enactment of customer orientation strategy has not been given enough scholarly attention. [28] suggests that brand legitimacy as value co-creation practices can prevent customers from engaging in undesirable behavior, such as purchasing and consuming unethical or illicit goods. Literature on brand legitimacy indicates that customers perceive unethical or illicit goods as neither socially responsible nor socially accountable [47]. Therefore, this present study seeks to examine the significant role of customers in granting legitimacy in the hospitality industry. While building a theoretical stance on social contract theory, the study theorized that COVID-19 preventive measures should be categorized as phrases of the social contract that bind together customers and hospitality organizations. In addition, the study seeks to examine how hospitality organizations can build legitimacy in situations of global pandemics such as COVID-19 through investing in customers as legitimacy-granted constituents. Thus, the study's findings are expected to help marketers and managers in the hospitality industry survive in the event of any global pandemic similar to COVID-19.

### COVID-19 preventive measures (CPM)

COVID-19 is the short form of the disease caused by the novel coronavirus, which was first discovered in Wuhan, China, in 2019 and has been constantly spreading throughout the world [61]. The Johns Hopkins Coronavirus Resource Center (2020) reported that up to the end of 2020, the world recorded and confirmed more than 64 million COVID-19 cases and more than 1.5 million deaths. Overall, scientists have confirmed that like other respiratory viruses, COVID-19 is spread to a great extent through human-to-human interaction, particularly physical contact that involves face-to-face conversation [61]. Thus, different countries adopt different mechanisms to slow down the rapid spread of the COVID-19 pandemic through approaches such as stay-at-home orders and, more importantly, emphasis on residents to reduce unnecessary human-to-human interaction or physical contact [39]. Despite mechanisms to reduce the rapid spread of COVID-19, several effects have already been identified in different industries, including hospitality and tourism. Evidence shows that the hospitality industry has been highly affected due to its highly interactive nature, involving human-to-human interactions [17].

In Tanzania, the first case of the COVID-19 pandemic was discovered in the Arusha region on March 16, 2020 [46]. This region is located in the northern part of Tanzania, and it is among the regions most endowed with natural tourist attractions [6]. Statistics from [42] indicate that due to COVID-19, Tanzania would expect the number of tourists to drop from 1,867,000 to 437,000, a decline of almost 76.6%. Subsequent to a drastic drop in tourists, it was estimated that revenue would also decrease from a projection of 2.7 trillion Tanzania shillings to 598 billion Tanzania shillings [42]. Overall, a significant drop in tourists is due to infection risk, which prevents tourists from visiting different countries as customers in the tourism industry [17]. More specifically, the Tanzanian hospitality industry was expected to lose more than 80% of its revenue by the end of 2020 [46]. Empirical evidence shows that the hospitality industry has been severely affected by the COVID-19 pandemic resulting in the majority of hotels and restaurants suspending their operations in order to reduce costs and contagion [45]. Because of the fact that most hotels and restaurants suspended their operations, the sector experienced a significant decrease in sales revenues and profits [45]. A report from the Tanzania National Bureau of Statistics (TNBS) indicates that hotel occupancy rates dropped from between 49% and 60% in 2019 to just 9% in mid-2020 [58]. In addition, the sudden suspension of operations at most hotels left a significant number of people jobless [45].

Similar to other countries in the world, during the post-COVID-19 pandemic, the government of Tanzania adopted several measures to revive the hospitality and tourism industries by using a phased approach [34]. These measures were taken due to a slow downward trend of COVID-19 cases worldwide. In addition, Tanzania has adopted prevention measures or guidelines by the World Health Organization (WHO) to assist hospitality organizations to operate safely [46]. Overall guidelines by WHO cover preventive measures in most critical areas, such as food safety, cleaning and sanitizing, employee health and hygiene, and social distancing [64]. Thus, COVID-19 preventive measures can be described as health and safety measures by WHO that suggest and highlight principles and guidelines to be followed to slow the spread of the pandemic. Given the interactive nature of the hospitality industry, COVID-19 brings a higher risk since the coronavirus can spread through respiratory droplets during oral communication or face-to-face communication [17]. Overall, COVID-19 prevention measures were found to be applicable in the hospitality industry because they provide fundamental principles for how human-to-human interaction should be practiced during the pandemic.

However, reviving the hospitality industry during the post-COVID-19 pandemic is inevitable because the industry employs a significant number of people and contributes significantly to GDP [46]. Furthermore, empirical evidence indicates that customers of hospitality organizations such as restaurants and hotels miss socializing with friends while also watching sporting events on the big screens in bars [61]. However, when different countries, including Tanzania, have taken measures to revive the hospitality industry during the post-COVID-19 pandemic, the empirical evidence suggests that customers differ significantly in predicting the role of preventive measures against COVID-19 on business operations [64]. This debate was also investigated by scholars such as [23]. The findings from [23] revealed that different people might develop different attitudes toward the role of COVID-19 preventive measures, as these business actions or practices might lead to the adoption of new lifestyles or perceptions. For instance, even though wearing masks has been scientifically confirmed to prevent the spread of COVID-19, there is a controversial debate about mask requirements [25]. Therefore, this study investigated customers’ perceived role of COVID-19 preventive measures in building brand legitimacy in the hospitality industry.

### Perceived brand ethicality (PBE)

Perceived brand ethicality represents customers’ perception of the brand as decent, responsible, honest, and accountable to its potential stakeholders [14]. According to the literature, brand ethicality has a theoretical root in ethical theory, which conceptualizes moral philosophy. Ethical theory suggests two potential bases for ethicality, namely deontology, representing rule-based and teleology, covering consequence-based [11]. Deontology concentrates on non-consequentialist ethics in which a person judges actions, whether they are right or wrong, in relation to specific higher moral standards or the law [10]. Teleology, on the other hand, encompasses consequentialist ethics, which considers the potential outcome and the extent to which good or bad will result from that action [54]. Thus, business organizations have started to leverage brand ethics as part of their strategic initiative through defining, differentiating, and sustaining their corporate brands in the turbulent marketplace [6]. Furthermore, the literature suggests that perceived brand ethicality covers customer ethical judgments, which are perceived to be the functions of both consequentialist (teleological) and non-consequentialist (deontological) ethical principles [14]. Therefore, customers evaluate brand ethicality from both streams of ethical theories.

Specifically, in a deontological context, customers evaluate brand ethicality by judging whether the hospitality organizations comply with environmental laws, financial laws, labor laws, etc. [11]. In addition, it covers the rule-based customer approach in the evaluation of moral norms, including integrity, transparency, fairness, honesty, etc. Also, ethical brands are perceived to ascribe human values of trust, empathy, and care toward their stakeholders due to anthropomorphic thinking [14, 53]. On the other hand, in a teleological context, customers evaluate brand ethicality by viewing the positive impact of the business organization’s practices. This consists of customers’ consequentialist approach, including corporate social responsibility, hands-on social engagement, and philanthropy [10]. The literature suggests that COVID-19 preventive measures in the hospitality industry, among other things, ensure customers build ethicality toward hospitality organizations in both consequentialist (teleological) and non-consequentialist (deontological) ethical principles [61]. For instance, in the consequentialist (teleological) approach, COVID-19 preventive measures intend to ensure consequences in terms of the health and safety of customers [67]. On the other hand, non-consequentialist (deontological), COVID-19 preventive measures should ensure customers develop ethicality toward the hospitality organization through the practices and actions of employees who demonstrate their ability to comply with acceptable moral standards and laws during pandemics [67].

#### H_1_

COVID-19 preventive measures influence perceived brand ethicality.

### Brand legitimacy (BRL)

Literature suggests that legitimacy is a socially constructed phenomenon that presents congruence between the behavior of a business organization and the assumedly shared beliefs of a particular social group (customers) [5, 32]. In this study, the term brand legitimacy is defined as the customers’ perception that the actions and practices of the brand are desirable and appropriate within a well-defined and acceptable socially constructive system of norms, beliefs, trust, values and definitions. Theoretically, brand legitimacy is constructed by both streams of ethical theories: consequentialist (teleological) and non-consequentialist (deontological) ethical principles. [49] argues that customers grant legitimacy to the brand when they perceive that there are shared and coincident behaviors, values, and beliefs. Overall, brand legitimacy offers a business organization credibility, moral authority, trust, and support from potential stakeholders, including customers. During pandemics such as COVID-19, brand legitimacy can ensure the hospitality organization accesses potential resources for their survival, including customer visitation and loyalty [67]. In a theoretical context, brand legitimacy can be conceptualized in three different forms: pragmatic brand legitimacy, moral brand legitimacy, and cognitive brand legitimacy.

### Pragmatic brand legitimacy (PBL)

Pragmatic brand legitimacy covers social acceptance that a constituent grants due to the practical benefits a business organization offers the constituent [67]. Overall, pragmatic brand legitimacy follows deontology principles that constitute brand ethicality. Pragmatic brand legitimacy has three aspects. First, it involves exchange dimensions, in which constituents support a business organization due to its expected practical benefits. Practical benefits include direct benefits to individual customers, societal and national welfare. Second, pragmatic brand legitimacy involves persuading dimensions, which cover how the business organization is responsive to the overall interests of constituents. Business organizations should consider the interests of their constituents beyond their own, as well as the interests of society and institutional environments where their constituents live. Third, pragmatic brand legitimacy involves disposition dimensions reflecting constituents' support since business organizations and constituents share values. Constituents grant legitimacy if they believe a business organization is looking out for their best interests [47]. Thus, pragmatic brand legitimacy covers whether consumption of a particular product is considered favorable for a person’s image in a given social setting and whether the ethical standards maintained by the reference group are similar to their own. It is therefore hypothesized that

#### H_2_

Perceived brand ethicality influence pragmatic brand legitimacy.

### Moral brand legitimacy (MBL)

Moral brand legitimacy presents a concern if the business organization is "doing the right thing" and whether, through doing the right things, a business organization promotes social well-being or social welfare [49]. It is social acceptance granted by a constituent after making judgments that the practices of business organizations are in line with the moral values of constituents [49]. Moral brand legitimacy involves consequential, procedural, structural, and personal aspects and hence is the result of the teleological principles of brand ethicality. The consequential dimensions hold the approval of a business organization's input and output [8]. The procedural aspect supports business organizations' procedures and methods [8]. Also, the structural part presents approval of the structural parameters of business organizations. In this context, constituents grant moral brand legitimacy when parameters are morally favored based on the moral codes of the constituents [49]. Lastly, the personal aspect rests on the personality of an individual, organizational leaders, and whether leaders of business organizations fit into the moral taxonomic classes of constituents. Moral legitimacy indicates whether individuals’ consumption behaviors align with the moral norms in force and if consumers of a certain product or service are perceived to represent morally sound or morally weak values [49]. It is therefore hypothesized that

#### H_3_

Perceived brand ethicality influence moral brand legitimacy.

### Cognitive brand legitimacy (CBL)

Cognitive brand legitimacy means gaining social acceptance granted by a constituent when appreciating the practices that position business organizations as a permanent and necessary part of their social setting [43]. It focuses on how the activities of the business organization are perceived to be ethical and meaningful in society. It is made up of necessity, permanence, inevitability, dependability, and knowledge of the business organization within society [44, 49]. Often cognitive brand legitimacy involves the degree to which a person's social settings accept or allow the purchase and consumption of a given product or service as essential and consider the nonexistence of such purchase or consumption inconceivable [49]. Cognitive legitimacy includes either affirmative support for a business organization or simply accepting the business organization as compulsory or inevitable within society [44]. It is related to opinions or ideas about the permanence of business organizations in society, and thus is the result of teleological principles of brand ethicality. Cognitive legitimacy is related to identifying the reasons behind decision-making, therefore facilitating understanding and delivering better solutions for different problems when they arise [49]. It is therefore hypothesized that

#### H_4_

Perceived brand ethicality influence cognitive brand legitimacy.

## Theoretical framework

### Social contract theory (SCT)

According to [18], social contract theory is a family of normative approaches to ethics that conceptualize how business organizations should respond when dealing with issues that have right and wrong implications. The theory emphasizes the conceptualization of specific ethical standards or norms related to ideas of right and wrong behavior shared within a social community [65]. A social community includes individuals capable of establishing specific norms of behavior that guide their interaction when sharing values, interests, goals, etc. Thus, if norms of behavior are consistent with overall moral standards, then these norms are accepted as ethical issues to be complied with all social community members. [16] argues that social contract theory is applied in marketing ethics in conceptualizing exchange themes. [18] suggests that in the context of social contract theory, business organizations deliver unique promises or benefits to customers in exchange for the unique and potential privileges of being able to exist and be profitable. Scholars such as [15] suggest that social contract theory becomes valid when a brand is perceived as a promise offered by a business organization to its customers. [16] used social contract theory to explain brand promises as indirect or inexplicit obligations in the contract, representing unwritten consumer rights to be fulfilled by the business organization.

**Fig. 1:**
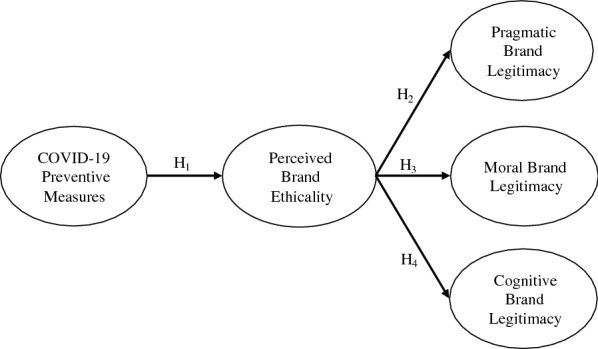
Theoretical model

In theory, brand promises establish specific ethical norms or standards that enforce the concept of right and wrong behavior between businesses and their customers [41]. Thus, when brand promises are consistent with overall moral standards, promises are absorbed as ethical norms to be observed by both business organizations and customers [68]. Thus, this study proposed that COVID-19 preventive measures should be perceived as brand promises that produce specific norms of behavior consistent with overall acceptable moral standards [41]. In pandemics such as COVID-19, preventive measures provide a theoretical and practical understanding of how business organizations should respond when dealing with matters that have right and wrong implications [41]. Social contract theory has been applied in this study to explain how COVID-19 preventive measures as brand promises can benefit customers in exchange for the unique and potential privileges to exist and build brand legitimacy. As presented in Fig. 1, the study used social contract theory to hypothesize a five-factor model that suggests that COVID-19 preventive measures would predict brand legitimacy (i.e., pragmatic brand legitimacy, moral brand legitimacy, and cognitive brand legitimacy) via perceived brand ethicality.

### Methods

This study used a cross-sectional survey research design in which data were collected at one given point in time across a sample population or a pre-defined subset. Through a cross-sectional research design, the topic under investigation was examined during a single instance with a defined starting point and ending point [57]. The design is more profitable in collecting data within a short period of time and is less expensive. In addition, the topic under investigation contains variables that do not change much due to the short period of data collection. The out site survey was conducted from June 2021 to August 2021. The study population was visitors who visited various hospitality organizations, including hotels and restaurants in Arusha and the Kilimanjaro region in Tanzania. The sample size of the study was determined using convenience or accidental sampling. The sampling technique is more suitable when the study population does not have a sampling frame. A total of 445 respondents were contacted, out of which 405 (91%) willingly filled out the questionnaire. This responses rate is above 50% as suggested by various scholars and researchers in social sciences researches. Overall sample size of 405 respondents meet the methodological requirements of multivariate analysis techniques, notably structural equation modeling [27].

### Measurement of variables

The measurement items in the survey were borrowed from already validated scales in COVID-19 preventive measures, perceived brand ethicality, and brand legitimacy. All measurement items were captured using 5-point Likert scales anchored at 1-strong disagree to 5-strongly agree [37, 38]. However, to ensure all measurement scale fit the study settings and methodological issues in the hospitality industry, modifications were made through rewordings, coding, and replacing phrases or statements as presented in Table 2. COVID-19 preventive measures were captured using five measurement items adopted from [61, 64]. Perceived brand ethicality was measured by adopting five measurement scale by [14, 33]. Brand legitimacy was measured using a measurement scale by [43, 49].

### Data analysis

#### Factor analysis

Prior to CFA and testing of proposed hypotheses, the instrument items were examined using principal component extraction with varimax rotation to evaluate construct validity. Additionally, the study employed the Kaiser–Meyer–Olkin (KMO) measure of sampling adequacy to make comparisons of the magnitude of the observed correlation coefficients. From a statistical viewpoint, the value of KMO should be greater than 0.5 to permit factor analysis. Thus, twenty-one questions associated with the five study’s constructs were factor analyzed by means of principal component analysis with varimax rotation. The results presented in Table 1 show that the factor analysis produced a value of the Kaiser–Meyer–Olkin (KMO) measure of sampling adequacy of 0.889. On top of that, the value of KMO has outcomes from Bartlett’s Test of Sphericity, demonstrating a Chi-square of 4624.591 at *df* = 210, and significant at 0.000. These results demonstrate sufficient evidence for the appropriateness of factor analysis.

**Table 1:** Factor analysis

Kaiser–Meyer–Olkin measure of sampling adequacy = 0.889						
Bartlett’s test of sphericity approx. Chi-square = 4624.591						
*df* = 210						
Sig. = .000						
Variables and measurement items	Initial Eigenvalues	1	2	3	4	5
1. COVID-19 preventive measures	7.376					
cpm1		0.679				
cpm2		0.602				
cpm3		0.741				
cpm4		0.706				
cpm5		0.502				
2. Perceived brand ethicality	2.737					
pbe1			0.638			
pbe2			0.553			
pbe3			0.719			
pbe4			0.728			
pbe5			0.638			
3. Pragmatic brand legitimacy	1.823					
pbl1				0.761		
pbl2				0.821		
pbl3				0.756		
4. Moral brand legitimacy	1.517					
mbl1					0.724	
mbl2					0.693	
mbl3					0.779	
mbl4					0.816	
5. Cognitive brand legitimacy	1.492					
cbl1						0.642
cbl2						0.724
cbl3						0.673
cbl4						0.707

Overall, the results of factor analysis yielded five factors, which accounted for a total of 69.5% of the variance for the entire set of study variables. Specifically, the first construct in this study is named “COVID-19 Preventive Measures,” with all five items’ loadings, which explains 15.5% of the variance. The second factor is labeled “Perceived Brand Ethicality,” with all five items’ loadings. Based on the results, this factor explains 15.16% of the variance. The third factor is “pragmatic brand legitimacy”, with three items’ loadings, explaining 14.13% of the variance. The fourth factor is called “moral brand legitimacy” with all four items’ loadings, and it explains about 13.45% of the variance. Finally, the last factor is “cognitive brand legitimacy” with all four items’ loadings. The results indicate that this factor explains 11.28% of the variance.

#### Common method bias

This study used the same method and self-administered or self-reported surveys to collect data from the same customers that pose the susceptible to common method bias. The study adopted procedural and statistical remedies as recommended by [35] to detect the existence of common method bias. Specifically, procedural remedies include reducing ambiguities in measurement items and providing assurance of anonymity and confidentiality to respondents. On the other hand, statistical remedies include using Harman’s single-factor analysis, in which all latent variables were loaded into Exploratory Factor Analysis (EFA) in fixed rotation [48]. A common method bias can occur when a single factor explains 50% of the variance [48]. However, in this study, a single factor explains only 35.1% of the variance, so common bias was not a concern.

### Evaluation of measurement model

Confirmatory Factor Analysis (CFA) using AMOS 21 was carried out to evaluate the compatibility test and psychometric properties of the measurement model. Overall, all goodness-of-fit statistics (*x*^2^/*df* = 2.555, GFI = 0.9, CFI = 0.938, TLI = 0.928, IFI = 0.939, AGFI = 0.9, RMSEA = 0.062, PNFI = 0.770, PCFI = 0.800) produced conventional standard output, which indicates the model fit well to the data [9, 26, 31, 40, 56]. In addition, the psychometric properties of specific latent variables and the reliability of measurement items were evaluated to check for convergent and discriminant validity, internal consistency, and reliability. The results in Table 2 suggest that standardized factor loadings preceded the threshold of 0.5 and were positively significant (*p* < 0.001) and with *R*^2^ > 0.20 suggested good individual item reliability and convergent validity [59]. Therefore, each indicator represents its respective latent variables that support the convergent validity of the measured items [21, 51]. In addition, other dimensions of psychometric properties, i.e., composite reliabilities (CR), Cronbach’s Alpha (*α*) Coefficients, and Average Variance Extracted (AVE), were considered. The CR and α presented in Table 2 exceeded 0.7 thresholds, suggesting internal consistency of measurement scales [51, 52]. In addition, as presented in Table 3, the value of AVEs > 0.5 thresholds supports the convergent validity of the measured items [60]. Discriminant validity was assessed using conditions and procedures suggested by [20]; the value of correlation between variables was compared with the corresponding AVEs value. The discriminant validity was supported since the value of correlation between variables was less than the correspondence square root of AVEs value [1]. Also, the value of Maximum Shared Variance (MSV) for each variable was less than its correspondence value of the square root of AVE.

**Table 2:** Reliability and validity of the measurement items

Constructs	Items	Factor loadings	*α*	CR
COVID-19 preventive measures			0.844	0.851
	This hospitality organization implement strict handwashing practices, including how and when to wash hands	0.723		
	This hospitality organization makes hand sanitizer readily available to guests	0.645		
	This hospitality organization is redesigning seating arrangements to ensure distancing between tables setups in dining areas	0.827		
	This hospitality organization update guests on a regular basis about necessary precautions and measures	0.816		
	This hospitality organization employees wear face-covering every moment when are in the workplace	0.629		
Perceived brand ethicality			0.860	0.864
	I believe this hospitality organization is a socially responsible company	0.733		
	I believe this hospitality organization will make a decision only after careful consideration of the potential positive or negative consequences for all those involved	0.668		
	I believe this hospitality organization respects moral norms	0.800		
	This hospitality organization is more beneficial for the welfare of the society than others	0.812		
	The way this hospitality organization behave is an example of how companies should be behaving in other countries in this situation	0.721		
Pragmatic brand legitimacy			0.856	0.858
	This hospitality organization activities benefit me	0.774		
	This hospitality organization activities have my community’s best interests at heart	0.858		
	This hospitality organization satisfies my needs and interests	0.820		
Moral brand legitimacy			0.889	0.891
	This hospitality organization is decent	0.787		
	This hospitality organization is wise	0.787		
	This hospitality organization is honest	0.834		
	This hospitality organization is trustworthy	0.868		
Cognitive brand legitimacy			0.842	0.846
	The service provided by this hospitality organization is well managed	0.712		
	Overall this hospitality organization actions and activities performed in the best possible manner	0.810		
	This hospitality organization is a necessary part of my community	0.750		
	I understand what this hospitality organization does and offers	0.770		

*α* Cronbach’s alpha, *CR* composite reliability

**Table 3:** Discriminant validity through Fornell–Larcker procedures

	AVE	MSV	MaxR(H)	1	2	3	4	5
1. MBL	0.672	0.403	0.896	**0.820**				
2. CPM	0.537	0.225	0.869	0.295	**0.733**			
3. PBE	0.561	0.403	0.871	0.635	0.342	**0.749**		
4. CBL	0.580	0.285	0.850	0.534	0.474	0.467	**0.761**	
5. PBL	0.669	0.267	0.864	0.511	0.212	0.517	0.270	**0.818**

Bolded value denotes square root of AVE

### Evaluation of structural model and hypotheses testing

In order to test the hypotheses presented in the hypothesized model, estimation and analysis of the structural model were done. Overall, all goodness-of-fit statistics (*x*^2^/*df* = 2.943, GFI = 0.9, CFI = 0.920, TLI = 0.909, IFI = 0.921, AGFI = 0.9, RMSEA = 0.069, PNFI = 0.779, PCFI = 0.811) yielded accepted scores, which suggests a perfect fit of the model to the data [26, 27, 31, 35]. The structural path coefficients estimates indicate that all hypothesized relationships proposed in the theoretical model get statistical support. Table 4 shows that perceived brand ethicality is influenced by COVID-19 preventive measures (*β* = 0.355; *t* = 6.120; *p* < 0.001), which supports hypothesis 1. In addition, the impact of perceived brand ethicality on pragmatic brand legitimacy is depicted (*β* = 0.474; *t* = 8.972; *p* < 0.001), which means hypothesis 2 is supported. Also, the results show that perceived brand ethicality affects moral brand legitimacy (*β* = 0.630; *t* = 11.049; *p* < 0.001), and therefore, hypothesis 3 is confirmed. Finally, the results reveal that perceived brand ethicality has a positive impact on cognitive brand legitimacy (*β* = 0.566; *t* = 8.474; *p* < 0.001) and thus, hypothesis 4 is approved.

**Table 4:** Hypotheses, path coefficients and *t*-statistics

Hypotheses	Path models	Path coefficients	SE	*t*-statistics	Support (yes/no)
H_1_	CPM → PBE	.355	.058	6.120***	Yes
H_2_	PBE → PBL	.474	.053	8.972***	Yes
H_3_	PBE → MBL	.630	.057	11.049***	Yes
H_4_	PBE → CBL	.566	.067	8.474***	Yes

***p < 0.001, Chi-square (*x*^2^)

*GFI* Goodness-of-Fit Index, *CFI* Comparative Fit Index, *TLI* Tucker–Lewis Index, *IFI* Incremental Fit Index, *AGFI* Adjusted Goodness-of-Fit Index, *RMSEA* Root Mean Square Error Approximation, *PNFI* Parsimony Normed Fit Index, *PCFI* Parsimony Comparative Fit Index

### Testing of mediation effects

The mediation effect of PBE in the relationship between CPM and MBL, PBL, and CBL was examined using four conditions for testing mediation recommended by Baron and Kenny (1986). *First*, the dependent variable should be influenced by the independent variable. Overall, the results fulfilled this condition as presented in Tables 5, 6, and 7 that MBL is affected by CPM (*ß* = 0.2228; *p* < 0.001), CPM affect PBL (*ß* = 0.1702; *p* < 0.001), and CPM affect CBL (*ß* = 0.4173; *p* < 0.001). *Second*, the independent variable should affect the mediator variable. As demonstrated in Tables 5, 6, and 7, this condition was achieved because CPM predict PBE (*ß* = 0.0.2655; *p* < 0.001). *Third*, the dependent variable should be affected by the mediator variable. Also, this condition was achieved as revealed in Tables 5, 6, and 7 as PBE influence MBL (*ß* = 0.5104; *p* < 0.001), PBE influence PBL (*ß* = 0.4445; *p* < 0.001), and PBE influence CBL (*ß* = 0.3597; *p* < 0.001). *Fourth*, the significance level between the independent and dependent variables when the mediator variable is introduced in the regression models can show a trivial change (indicating partial mediation) or turn into insignificance (indicating full mediation). The results demonstrated in Tables 5, 6, and 7 indicate that partial mediation was achieved in the relationship between CPM → PBE → MBL (*ß* = 0.0873; *p* < 0.05) and CPM → PBE → CBL (*ß* = 0.3218; *p* < 0.05) whereby the level of significance fell slightly but not insignificantly. However, full mediation was achieved in the relationship between CPM → PBE → PBL (*ß* = 0.0521; *p* > 0.05).

**Table 5:** Sobel test statistics

Mediation path	Path coefficient	Sobel statistics	Supported (yes/no)
C_1_:CPM → MBL	0.2228	5.4926***	Yes
C_2_:CPM → PBE	0.2655	6.3142***	Yes
C_3_:PBE → MBL	0.5104	12.5019***	Yes
C_4_:CPM → PBE → MBL	0.0873	2.4159**	Yes

*C* condition

***p value < 0.001, **p value < 0.05

**Table 6:** Sobel test statistics

Mediation path	Path coefficient	Sobel statistics	Supported (yes/no)
C_1_:CPM → PBL	0.1702	3.8596***	Yes
C_2_:CPM → PBE	0.2655	6.3142***	Yes
C_3_:PBE → PBL	0.4445	9.3856***	Yes
C_4_:CPM → PBE → PBL	0.0521	1.2441*	Yes

*C* condition

***p value < 0.001, *p value > 0.05

**Table 7:** Sobel test statistics

Mediation path	Path coefficient	Sobel statistics	Supported (yes/no)
C_1_:CPM → CBL	0.4173	8.8634***	Yes
C_2_:CPM → PBE	0.2655	6.3142***	Yes
C_3_:PBE → CBL	0.3597	6.8009***	Yes
C_4_:CPM → PBE → CBL	0.3218	6.8766**	Yes

*C* condition

***p value < 0.001, **p value < 0.05

## Results and discussion

The current study aimed to examine the perceived role of COVID-19 preventive measures in building brand legitimacy with a mediating role of perceived brand ethicality. It is among the few endeavors that attempt to extend brand management knowledge during turbulence resulting from pandemics similar to COVID-19. The study theorizes that in situations of pandemics such as COVID-19, customers expect brands to behave in manners that demonstrate their concern for social norms and moral standards. Overall, the study intends to propose that hospitality organizations, particularly those from the hospitality industry, should take a dramatic shift to strategies that replace traditional business strategies. The results indicate that COVID-19 preventive measures influence various forms of brand legitimacy with the mediating role of perceived brand ethicality. The results suggest that COVID-19 preventive measures in the hospitality industry could help hospitality organizations such as hotels, restaurants, etc., elicit the feeling that they are concerned with society's social norms and moral standards. It is important to note that hospitality organizations could strengthen perceived brand ethicality and ultimate brand legitimacy by not taking advantage of the COVID-19 pandemic to maximize profits [47]. The results present the need for hospitality organizations to enhance customers’ well-being or welfare to build sustainable business practices during pandemics. It is widely accepted that perceived brand ethicality, i.e., ethical practices by hospitality organizations, could help build brand legitimacy. These results align with [64] that COVID-19 preventive measures are important renovated strategies that motivate customers to dine out. Overall, customers consider hospitality organizations that implement COVID-19 preventive measures socially and morally responsible. It presents a more customer-centric service organization, treating customers as the central focus for the survival of the organization.

Furthermore, the results demonstrate the mediating role of perceived brand ethicality in the relationship between COVID-19 preventive measures and brand legitimacy. The results indicate that a higher level of perceived brand ethicality motivates customers in the hospitality industry to build brand legitimacy. Customers consider COVID-19 preventive measures as ways to define a brand that acts ethically, and therefore, ethical practices could have several benefits for customers [22]. During the COVID-19 pandemic, perceived ethical practices could offer several benefits, including avoiding severe acute health effects, including death. In addition, the results suggest that customers legitimize the brand in a moral context when hospitality organizations behave ethically to the extent of building trustworthiness, honesty, wisdom, decent character, etc. [47] supports these findings by arguing that ethical behaviors are vital for building corporate legitimacy in pandemics and enhancing business performance. COVID-19 preventive measures positively impacted perceived brand ethicality, motivating customers to build trustworthiness, honesty, wisdom, and decent character as part of moral brand legitimacy. Thus, the perceived ethical practices of the brand can increase customers’ trust and righteousness toward hospitality organizations in the hospitality industry. These findings support the argument of [48] that brand trust is an important driver that accelerates the strength of COVID-19 preventive measures as determinants of customers’ intention to dine out in restaurants.

On the other hand, perceived brand ethicality that involves ethical practices of hospitality organizations can influence customers to build cognitive brand legitimacy. This implies that COVID-19 preventive measures motivate customers to perceive the practices of hospitality organizations as ethical practices that ultimately influence them to build cognitive brand legitimacy. In the milieu of this study, cognitive legitimacy covers customers’ feelings that the hospitality organizations are well managed through following well-known procedures, operations, and practices. Overall, practices indicate that during the pandemic era, such as COVID-19, prevention measures should also focus on helping customers gain knowledge about the brand and its practices. Therefore, the results indicate that proper COVID-19 preventive measures should focus on instilling perceived brand ethicality, i.e., ethical practices that ultimately influence customers to grant legitimacy to the brand. These findings support the argument by [12], who argues that, in the aftermath of COVID-19, tourists’ outbound travel behavior and destination attachment are shaped by corporate social responsibility and the perceived response efforts. Furthermore, health preventive behavior and overall destination attachment are crucial drivers of various tourists' outbound travel behaviors. In addition, the results are in line with [64], who revealed the mediating role of brand trust in the relationship between the perceived importance of preventive measures and customers’ intention to dine out. In addition, the relationship between the perceived importance of preventive measures and brand trust is moderated by the positive country of origin effect.

## Conclusion

### Theoretical

Based on the study findings and their discussion, the study presents conclusions from both theoretical and managerial perspectives. In a theoretical context, the study provides conclusions based on the study's theoretical contribution. On the other hand, the study offers a conclusion that focuses on spotting the relevance and importance of the study to managers of hospitality organizations. In a theoretical context, this study extends to the body of literature by examining the role of COVID-19 prevention measures in building brand legitimacy with a mediating role of perceived brand ethicality. Several studies on brand legitimacy have been conducted; however, there is little evidence on the antecedents of brand legitimacy during pandemics, particularly in highly interactive industries such as the hospitality industry. In addition, although literature suggests that customers grant brand legitimacy, scant evidence exists in the hospitality industry regarding customers' role in granting legitimacy during global pandemics similar to COVID-19.

Therefore, the present study contributes to the body of literature by approving the mediation role of perceived brand ethicality in the relationship between COVID-19 preventive measures and brand legitimacy. On top of that, numerous studies about infectious diseases, including Severe Acute Respiratory Syndrome (SARS), have been conducted from the hospitality and tourism industry perspective. However, the study on global pandemics such as COVID-19 requires special attention to examine its impact on brand-building behavior and other behavioral intentions. Literature indicates that COVID-19 is a global pandemic that affected the hospitality industry at a global level and not only in specific countries like SARS. Therefore, this study examines the interplay of COVID-19 preventive measures, perceived brand ethicality, and brand legitimacy in the form of relationships that have not been well examined in previous studies, notably in the hospitality industry. With the help of social contract theory, this study confirms that perceived brand ethicality strengthens the relationship between COVID-19 preventive measures and brand legitimacy. Thus, during pandemics similar to COVID-19, different preventive measures are perceived as social norms and practices that can help customers define the wrongs and right practices of service organizations during encounters.

### Practical

In addition, in a managerial or practical context, the study poses conclusions that could benefit the managers of hospitality organizations. Overall, the study provides a policy and strategic framework for managers of hospitality organizations in managing risk related to COVID-19 and other similar pandemics. Practical evidence indicates that, even though the COVID-19 pandemic has an acute negative impact on highly interactive industries such as the hospitality industry, some hospitality organizations have not been able to comply with WHO guidelines and procedures for fighting and combating the COVID-19 pandemic. Therefore, it is recommended that managers in hospitality organizations adopt guidelines and preventive measures as renovated strategic plans and marketing strategies build a competitive edge during pandemics. Specifically, COVID-19 preventive measures should be embraced as potential dimensions for building renovated positioning strategies. In other words, since customers use COVID-19 preventive measures to define the wrong and right practices of hospitality organizations, managers should consider preventive measures as part of value propositions. As a result of COVID-19 preventive measures, marketers can occupy space in customers' minds by introducing specific messages about ethical practices that take into account the welfare and well-being of customers.

Furthermore, to survive during pandemics such as COVID-19, managers must incorporate some elements of health and safety into the mission of hospitality organizations. Overall, to build brand legitimacy through perceived brand ethicality, hospitality organizations should ensure preventive measures become part of the mission representing hospitality organizations in the marketplace. Overall, mission presents the reason for the existence of particular hospitality service organizations. Thus, during pandemics such as COVID-19, the reasons for the existence of hospitality organizations should be beyond profit-making and should instead ensure the social welfare or well-being of current and potential customers. Additionally, preventive measures should be incorporated into the overall marketing efforts of hospitality organizations that seek to build corporate reputations. Overall, marketers of hospitality organizations can enhance the possibility of customers granting legitimacy through regular and intense communication within institutional environments. Therefore, all marketing communication efforts or campaigns should focus on presenting hospitality organizations as a place where the health and safety of customers are highly valued. Furthermore, it should inform customers that preventive measures have been integrated into the renovated Corporate Social Responsibility (CSR) policy, strategy, and practices. Overall, the extent to which hospitality organizations are highly responsible and accountable for their customers and society should be communicated in programs and outlets such as advertising, digital marketing platforms, websites content, sponsorships, etc. [29].

On top of that, hospitality organizations should put in place operational standards that provide guidelines to service providers (staff, employees, etc.) during encounters with customers. In the lens of social contract theory, these operational standards assume attributes such as brand promises that bind customers and service providers. Thus, customers and service providers should be encouraged to comply with and adhere to all operational standards to build brand legitimacy. Within the context of this study, operational standards can include wearing a facemask, hand sanitizer, sitting arrangements that consider social distancing, and regular orientations with guests about precautions against COVID-19. For instance, regular orientations should inform customers about right and wrong consumption and right and wrong practices or actions during pandemics in the hospitality industry. The aim is to ensure customers become more aware of the pandemic, inspiring them to comply with preventive measures.

Furthermore, since the hospitality industry is highly interactive, COVID-19 preventive measures should be used as guiding procedures and standards during service encounters. In addition, the interactive nature of the hospitality industry influences customers to evaluate wrong and right practices through the behavior of service providers, i.e., employees or staff. This argument is supported by the idea that, given the salient features of services (e.g., inseparability and variability, homogeneity, etc.), customers evaluate the quality of service through the behavior of service providers, i.e., employees or staff. In this regard, service organizations, i.e., hospitality organizations, are just abstract entities represented by employees or staff. Therefore, customers, as legitimacy-granting constituents, can be influenced to grant legitimacy to the brand due to the behavior of employees or staff. The study demonstrates that when preventive measures are adopted as guiding procedures and standards during encounters, they can influence customers to consider them as ethical practices that trigger brand legitimacy. Based on this argument, it is crucial for marketers to ensure all procedures encourage service providers (i.e., employees or staff) to demonstrate a sense of willingness and commitment to ensure customers' health and safety during the encounter as a precondition for building perceived brand ethicality.

### Limitations and recommendations for future research

Although the present study has a potential contribution to the body of knowledge about branding during pandemics, few limitations have been observed that offer room for future research. First, the study was conducted in the Tanzanian hospitality industry, and hence, the findings should be generalized with caution. This is because, given the nature of the hospitality industry, cultural differences and contexts can offer a different picture of the subject under study when conducted in other countries. Based on this, the study recommended future research in different contexts and cultural settings to solidify the study's findings. In addition, a comparative study can be conducted to establish differences in COVID-19 preventive measures and their consequences across different hospitality industries. Furthermore, a longitudinal research design could supplement the weakness of the cross-sectional design used in this study. Specifically, future research can examine changes in responses to COVID-19 preventive measures over time through longitudinal research design. When the intensity of a pandemic decreases, customers may respond differently to these preventive measures, and hence, different ways of managing its consequences may be necessary. Finally, the study uses a quantitative approach, limiting the possibility of getting the qualitative side of the story. Then, future research should use a qualitative method to get a more realistic picture of the subject matter see [30].

## Data Availability

Not applicable.

## Abbreviations

MNRT: Ministry of Natural Resources and Tourism
WHO: World Health Organization
